# Occurrence of the Ochratoxin A Degradation Product 2′R-Ochratoxin A in Coffee and Other Food: An Update

**DOI:** 10.3390/toxins11060329

**Published:** 2019-06-08

**Authors:** Franziska Sueck, Vanessa Hemp, Jonas Specht, Olga Torres, Benedikt Cramer, Hans-Ulrich Humpf

**Affiliations:** 1 Institute of Food Chemistry, Westfälische Wilhelms-Universität Münster, Corrensstraße 45, 48149 Münster, Germany; f_suec01@uni-muenster.de (F.S.); vhemp@web.de (V.H.); jonasspecht@uni-muenster.de (J.S.); 2 Laboratorio Diagnostico Molecular S.A, Guatemala City, Guatemala; otorres@dxmolecular.com; 3 Centro de Investigaciones en Nutrición y Salud, Guatemala City, Guatemala

**Keywords:** Ochratoxin A, 2′R-ochratoxin A, 14(R)-ochratoxin A, coffee, degradation, processing, roasting, modified mycotoxins, masked mycotoxins

## Abstract

Food raw materials can contain the mycotoxin ochratoxin A (OTA). Thermal processing of these materials may result in decreased OTA levels but also in the formation of the thermal isomerization product 2′R-ochratoxin A (2′R-OTA). So far, only 2′R-OTA levels reported from 15 coffee samples in 2008 are known, which is little when compared to the importance of coffee as a food and trading good. Herein, we present results from a set of model experiments studying the effect of temperatures between 120 °C and 270 °C on the isomerization of OTA to 2′R-OTA. It is shown that isomerization of OTA starts at temperatures as low as 120 °C. At 210 °C and above, the formation of 25% 2′R-OTA is observed in less than one minute. Furthermore, 51 coffee samples from France, Germany, and Guatemala were analyzed by HPLC-MS/MS for the presence of OTA and 2′R-OTA. OTA was quantified in 96% of the samples, while 2′R-OTA was quantifiable in 35% of the samples. The highest OTA and 2′R-OTA levels of 28.4 µg/kg and 3.9 µg/kg, respectively, were detected in coffee from Guatemala. The OTA:2′R-OTA ratio in the samples ranged between 2.5:1 and 10:1 and was on average 5.5:1. Besides coffee, 2′R-OTA was also for the first time detected in a bread sample and malt coffee powder.

## 1. Introduction

**Figure 1:**
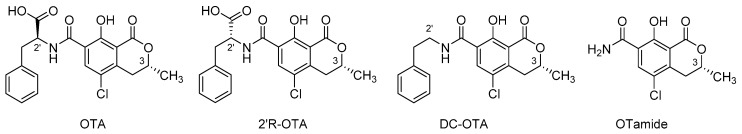
Structures of ochratoxin A (OTA), its thermal isomerization product 2′R-ochratoxin A (2′R-OTA), and the degradation products 2′-decarboxy-ochratoxin A (DC-OTA) and ochratoxin α-amide (OTamide).

The mycotoxin ochratoxin A (OTA, Figure 1) can be found in a broad spectrum of food raw materials infested with fungi of the genera *Aspergillus* and *Penicillium* as well as in food products derived from these commodities [1]. Considering exposure within the European Union, cereals and cereal products, such as pasta, bread, and beer, are the most relevant OTA sources due to the high consumption rates of these food items. In Germany, for instance, these products are on average responsible for about 67% of the OTA intake in adults. Besides this, approximately 12% of the total OTA exposure occurs from coffee drinking and a further 6% from cocoa, 6% from meat, and 5% from wine [1].

Most food items undergo food processing, such as milling, baking, roasting, frying, or fermentation. All of these steps can have an impact on the mycotoxin burden of the product, for instance by physical removal or chemical modification [2,3]. To what extent mycotoxins are modified during these processing steps depends on the parameters but also on the chemical structure of the compound. Chemical modifications can result in the formation of mycotoxin conjugates, such as the deoxynivalenol (DON) glucosides formed during the fermentation of dough [4,5,6], binding to food-matrix components as shown for fumonisins [7], or the formation of degradation products, such as the norDON series [8].

Depending on the commodity, OTA undergoes different types of processing, such as baking, extrusion cooking, roasting, and fermentation. The reduction of OTA levels during these processes has been found to range between no impact and almost a 100% decrease [3,9,10,11,12]. A strong OTA decrease was observed during coffee roasting, where up to a 90% reduction was reported in most of the studies [13,14,15,16,17]. On the other hand, in some other coffee-roasting experiments, only a slight reduction of less than 12% was noted [18,19]. Several efforts have been made to identify the chemical reaction that leads to lower OTA levels in roasted coffee. Bittner et al. (2013) showed that OTA binds to polysaccharides of the coffee bean during roasting [20]. The high temperatures present during coffee roasting also lead to a decarboxylation to form decarboxy-ochratoxin A (DC-OTA) as well as to racemization of the phenylalanine moiety to yield 2′R-ochratoxin A (2′R-OTA, previously reported as 14(R)-OTA) and to the formation of ochratoxin α-amide (OTamide) as shown in Figure 1 [20,21,22]. A quantitative analysis of 15 coffee samples from the German market indicated that DC-OTA is only formed in minor amounts, while the concentration of 2′R-OTA was up to 0.63 µg/kg. A ratio of OTA and 2′R-OTA lower than 4:1 was determined [21]. OTamide was not analyzed in this study.

OTA has been shown to be nephrotoxic, hepatotoxic, teratogenic, and immunotoxic in various species and was classified by the International Agency for Research on Cancer (IARC) as possibly carcinogenic to humans (group 2B) in 1993 [23,24]. The mode of action is still not clear and controversially discussed [25,26]. In comparison to OTA, only very little information on the toxicity of the degradation product 2′R-OTA is available: 2′R-OTA shows in a Cell Counting Kit-8 (CCK-8) assay a 10-fold lower cytotoxic effect on IHKE-cells compared to OTA [21,27]. Several studies have shown that OTA is detectable in blood of humans and animals [28], with human plasma concentrations ranging roughly between 0.05 ng/mL and 5 ng/mL [29,30]. The thermal isomerization product 2′R-OTA is also of relevance, as it was recently found in humans in comparable levels as OTA [31].

Little is known on the kinetics of the isomerization of OTA to 2′R-OTA, and the minimum temperature required for the thermal conversion of OTA to 2′R-OTA has not been elucidated [21]. Depending on these data, bakery products and breakfast cereals might also be potential sources of 2′R-OTA exposure.

Herein, we report the analysis of 51 coffee samples from Germany, France, and Guatemala to extend the database on 2′R-OTA levels in food. Furthermore, the kinetics of the isomerization of OTA to 2′R-OTA were studied in a model system covering temperatures between 120 and 270 °C. As isomerization of OTA to 2′R-OTA has already been observed at 120 °C, further thermally treated food samples were analyzed on the occurrence of OTA and 2′R-OTA.

## 2. Results

### 2.1. Degradation of OTA in Model Heating Experiments

**Figure 2:**
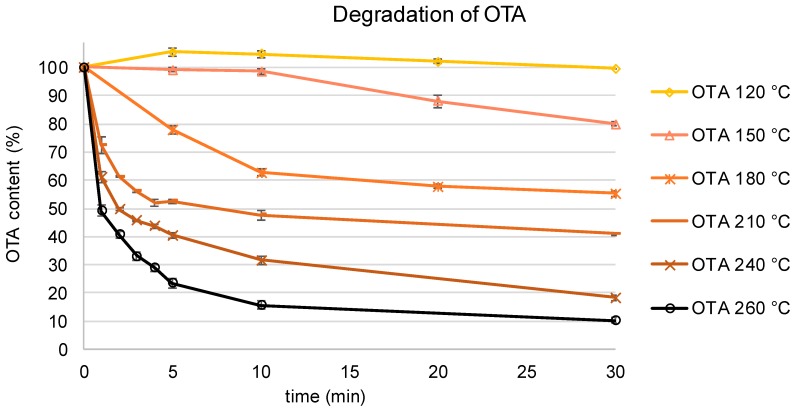
Degradation curves of ochratoxin A (OTA) at temperatures between 120 and 260 °C for 1–30 min.

**Figure 3:**
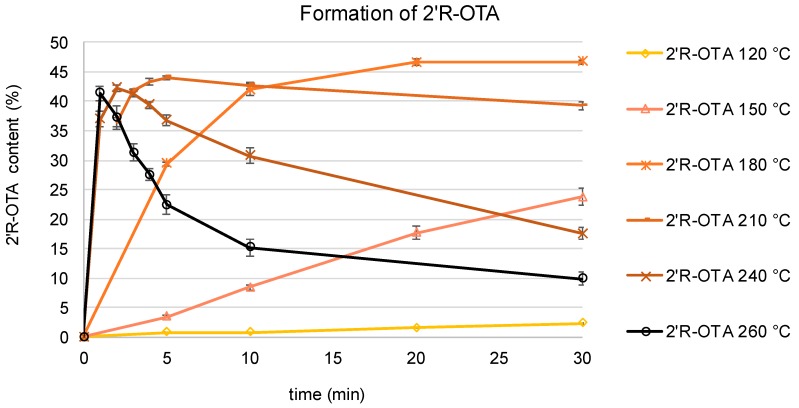
Formation of 2′R-ochratoxin A (2′R-OTA) during heating of OTA at temperatures between 120 and 260 °C for 1–30 min.

The isomerization of OTA to 2′R-OTA as well as the formation of other degradation products were studied in model heating experiments. To that end, OTA was heated at temperatures of 120, 150, 180, 210, 240, and 260 °C without solvent for 1 to 30 min, respectively. The observed degradation curves of OTA are shown in Figure 2. Subsequent analysis by HPLC-FLD for the known degradation products 2′R-OTA and DC-OTA resulted in the curves shown in Figure 3. At the lowest tested temperature of 120 °C, OTA remained almost stable over the entire heating period with only 3% 2′R-OTA formed within 30 min. After the same time, but heating at 150 °C, approximately 20% of OTA was converted to 2′R-OTA. At 180 °C, a fast racemization of OTA towards 2′R-OTA was observed with an equilibrium between both compounds reached after approximately 20 min. Above this temperature, racemization of OTA towards 2′R-OTA was achieved after 1 to 5 min of heating, followed by further degradation of both diastereomers. After 30 min at 210, 240, and 260 °C, only 80%, 35%, and 20%, respectively, of the sum of OTA and 2′R-OTA were detectable. Screening for DC-OTA and OTamide revealed only small quantities of less than 1% (data not shown), and were not further considered.

### 2.2. Coffee Powder: Current Situation of OTA and 2′R-OTA Content

**Figure 4:**
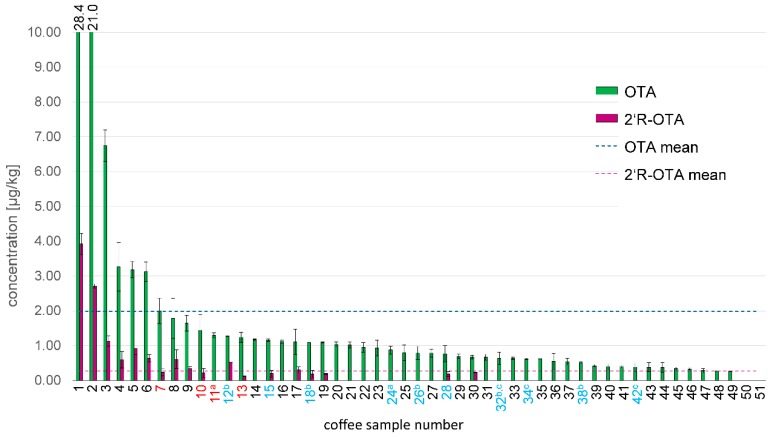
OTA and 2′R-OTA contents of the coffee powder samples from Germany (red), Guatemala (black), and France (blue). ^a^ decaffeinated, ^b^ espresso, ^c^ organically grown. The standard deviation is given for duplicate analysis.

The published dataset on 2′R-OTA contamination in food and the OTA:2′R-OTA ratio is limited to data from the analysis of 15 coffee samples in 2008 [21]. Therefore, in order to extend the database and to evaluate the current situation, we analyzed in total a set of 51 coffee samples from France, Germany, and Guatemala. Among the 14 commercially available roasted coffee samples from the French and German market, five were categorized as espresso powder and nine samples as coffee powder packages. Three of the coffee samples were labelled as organically grown and two as decaffeinated. The results are presented in Figure 4. In all coffee samples except two from Guatemala, OTA was quantifiable in a range between 0.26 and 28.4 µg/kg with a mean contamination of 1.98 µg/kg. No sample from Europe, but three samples from Guatemala (Figure 4, coffee samples 1-3) showed OTA levels above the legal limit of 5 µg/kg OTA set by the European Union [32]. Among these three, two samples exceeded the limit by factors of 5 and 4, respectively.

In two decaffeinated coffee samples (Figure 4, samples 11 and 24), OTA concentrations of 1.30 and 0.88 µg/kg were detected. Interestingly, the three analyzed organic coffee powders, including one espresso sample (Figure 4, samples 32, 34, and 42), with OTA concentrations of 0.64, 0.62, and 0.38 µg/kg were among the coffee samples with the lowest detected OTA levels. The four other analyzed espresso samples (Figure 4, samples 12, 18, 26, and 38) contained 1.27, 1.09, 0.79, and 0.51 µg/kg OTA, which was comparable to the OTA contents of the other coffee samples from Europe.

The isomer 2′R-OTA was quantifiable in 18 of the 51 coffee samples (35%) with a mean concentration of 0.27 µg/kg. The highest 2′R-OTA level of 3.9 µg/kg was determined in the coffee sample from Guatemala containing 28 µg/kg OTA (Figure 4, sample 1). Among the European coffee samples, an espresso coffee powder (Figure 3, sample 12) with a 2′R-OTA concentration of 0.52 µg/kg and an OTA level of 1.27 µg/kg showed the highest 2′R-OTA contamination and, furthermore, the highest OTA:2′R-OTA ratio of 2.5:1. However, there was no indication that espresso coffee production favors 2′R-OTA formation, as the other analyzed espresso coffee samples contained no, or a low amount of, 2′R-OTA and showed OTA:2′R-OTA ratios between 4:1 and 10:1. In both decaffeinated coffee samples (Figure 3, samples 11 and 24), the 2′R-OTA concentration was below the limit of quantification (LOQ). Taking all coffee samples into account that were positive for 2′R-OTA, a OTA:2′R-OTA ratio between 10:1 and 2.5:1 with an average OTA:2′R-OTA-ratio of 5.5:1 was observed.

### 2.3. Evaluation of Other Thermally Processed Food Materials as Sources of 2′R-OTA Exposure

The data from the heating experiment with pure OTA suggest that, at a comparatively low temperature of 120 °C, a slow isomerization of OTA to 2′R-OTA can be observed. Such temperatures can be reached during different kinds of baking processes, and the conditions might be sufficient for the formation of significant quantities of 2′R-OTA. To that end, different thermal processed food samples were screened (Table 1). The choice of the analyzed food samples was based on thermal processing conditions and the overall contribution to OTA exposure [1]. Consequently, 30 samples of cocoa (8) and cereal products (22) were analyzed for OTA and 2′R-OTA. Details on the samples are given in Table 1.

Generally, OTA contamination of the analyzed food samples was low, resulting in only two samples with OTA levels above the LOQ and 13 samples above the limit of detection (LOD). However, despite the low OTA contamination, six food samples were found to be positive for 2′R-OTA. In particular, coffee surrogates, such as instant malt coffee powder and malt coffee powder, were all found to be positive for this compound. With an OTA concentration of 0.62 ± 0.04 µg/kg and a 2′R-OTA concentration of 0.22 ± 0.02 µg/kg, comparable racemization rates as for roasted coffee were observed. Additionally, one bread sample (pumpernickel) contained detectable amounts of 2′R-OTA. Pumpernickel is a long-term heated bread. To produce this type of bread, a loaf of rye is baked for 16–24 h at an oven temperature of approximately 110 °C.

## 3. Discussion

The thermal instability of the mycotoxin OTA has been reported for several food processing technologies. However only for roasted coffee have the degradation products 2′R-OTA and DC-OTA been described and quantified so far, and in a limited number of samples. In this study, it was shown in model heating experiments that a slow racemization of OTA to 2′R-OTA occurs at temperatures as low as 120 °C. Above these temperatures, a fast racemization of OTA was observed, with 25% 2′R-OTA being formed after 30 min at 150 °C, after 5 min at 180 °C, and after 1 min or less at 210 °C and above. Longer heating periods at temperatures of 240 °C and above resulted in a fast degradation of OTA and 2′R-OTA, which can be explained by other reactions, such as pyrolysis or polymerization. Compared with previously reported data, these results confirm the importance of 2′R-OTA as the main OTA degradation product. A slow conversion of OTA to 2′R-OTA occurring at temperatures as low as 120 °C has not yet been reported [21,22].

Thus, food samples, processed at temperatures far lower than those applied for coffee roasting, might be additional sources of 2′R-OTA exposure. However, to date, 2′R-OTA has only been detected in blood samples from 34 coffee drinkers in concentrations between 0.021 and 0.411 ng/mL (mean: 0.21 ± 0.066 ng/mL) compared to 0.071–0.383 ng/mL (mean: 0.11 ± 0.093 ng/mL) for OTA. An average OTA:2′R-OTA ratio of 2:1 was determined and in some cases, the concentration of 2′R-OTA even exceeded that of OTA. No 2′R-OTA was detected in the set of 14 samples from non-coffee drinkers, suggesting that 2′R-OTA is predominantly present in roasted coffee. Nevertheless, the number of non-coffee drinkers participating in that study was low, and no correlation between the 2′R-OTA levels and overall coffee consumption was observed, making further sources of 2′R-OTA plausible [31,33].

Screening of a set of 51 coffee samples confirmed the role of roasted coffee as the key source of 2′R-OTA exposure, with 2′R-OTA levels of up to 3.9 µg/kg in highly contaminated coffee from Guatemala and up to 0.52 µg/kg in European coffee samples. No systematic differences between espresso coffee and other coffee with respect to the 2′R-OTA levels could be observed. For decaffeinated coffee, moderate OTA levels were detected, but no 2′R-OTA in concentrations above the LOQ. Although no information on the OTA content before roasting was available, the reported reductive effect of decaffeination on OTA levels seems to be limited [34]. These results are in good agreement with previously reported 2′R-OTA levels but also show that, in certain samples, such as one specific espresso powder, relatively high 2′R-OTA levels and OTA:2′R-OTA ratios as low as 2.5:1 can occur. In a previous study, for roasted coffee, OTA:2′R-OTA ratios between 10:1 and 4:1 (mean: 5:1) were determined from a set of 15 coffee samples. The variation of these ratios between different coffee samples also suggested a dependency of 2′R-OTA formation on the roasting process. Traditionally, green beans are roasted for between 8 and 20 min at temperatures between 160 and 240 °C using a drum roaster, a hot-air roaster, or a combination of both systems [35]. Oliveira et al. (2013) reported a correlation between roasting level and OTA degradation, while Castellanos-Onorio et al. (2011) observed for the two roasting techniques (drum roaster and hot-air roaster) a similar OTA reduction. In both cases, the formation or 2′R-OTA was unfortunately not studied [16,17]. In contrast to the traditional temperature regimes, to increase throughput, some companies have established a coffee-roasting process based on high temperatures of around 400 °C and roasting times of less than one minute [36]. 

To confirm the results from the model experiments, other thermally processed food samples, known to contribute to OTA exposure, were analyzed for the presence of 2′R-OTA, the most abundant thermal degradation product of OTA. Besides coffee, quantities of 2′R-OTA were detected in malt coffee as well as in traditionally baked rye bread (Pumpernickel). The latter is of special importance as it confirms the results from the model experiments, showing that OTA can be converted to 2′R-OTA at low temperatures between 100 and 120 °C. However, it has to be considered that this type of bread has a minimum baking time of 16 h, which makes a comparison with other bread and bakery products rather difficult. Other analyzed food samples contained no detectable amounts of 2′R-OTA but were also low for OTA.

The most relevant sources of OTA exposure are cereals, followed by coffee, cocoa, meat, and wine. For the cereal products biscuits and muesli, baking times are usually short and temperatures inside the product are mostly at 100 °C or below [37]. For roasted and expanded cereals, the situation is different; however, due to the low availability of industrially processed and naturally contaminated products, we were not able to prove whether a racemization of OTA during these processes may occur. Considering other typical food-processing procedures, wine can be excluded as potential source of 2′R-OTA due to low fermentation and processing temperatures. In the case of meat, temperatures above 100 °C are rarely reached in the inner part of the product (e.g., sausage). A different situation might be the manufacturing of canned meat, such as corned beef, where temperatures of up to 121 °C are applied for sterilization. Here, additional studies investigating the racemization of OTA in aqueous solutions are necessary. Thus, there is a need for more data on the occurrence of 2′R-OTA in these foods and on the toxic properties of this compound to allow for an adequate risk assessment.

## 4. Materials and Methods

Methanol (MeOH), acetonitrile (ACN), and toluene were obtained in gradient grade from Fisher Scientific (Schwerte, Germany). NaCl, formic acid, hexane, and Na_2_HCO_3_ were in pro analysi (p.a.) quality from Merck KGaA (Darmstadt, Germany). Potato dextrose agar (PDA), potato dextrose broth, and KH_2_PO_4_ were from Carl Roth (Karlsruhe, Germany), Xylene in p.a. quality was obtained from Honeywell (Seelze, Germany), NaHCO_3_ in p.a. quality was obtained from Grüssing (Filsum, Germany), and KCl in p.a. quality was obtained from VWR (Langenfeld, Germany). Purified water of ASTM type 1 quality was prepared with a Purelab Flex 2 system from Veolia Water Technologies (Celle, Germany).

### 4.1. Biosynthesis of Standards

OTA was isolated from cultures of *Aspergillus westerdijkiae* BFE 1115, provided by the Max Rubner Institute (Karlsruhe, Germany), which were activated for 2 days in liquid potato dextrose broth at room temperature and then incubated for two weeks at 27 °C on autoclaved durum wheat adjusted to a water content of 62.5% (*w*/*w*) and supplemented with 2.5% sodium chloride (*w*/*w*). OTA was extracted from the durum wheat cultures using tBME containing 0.5% formic acid, purified by liquid–liquid extraction with water at different pH levels and silica column chromatography with the solvent system toluene/tBME/formic acid (8/1.5/0.5 (*v*/*v*/*v*)). Finally, residual impurities were removed by crystallization from xylene/hexane (7/3 (*v*/*v*)). The purity of OTA was >99% as determined by HPLC-UV (220 nm) and NMR. 2′R-OTA, DC-OTA, OTα-amide, d_5_-OTA, and d_5_-2′R-OTA were prepared in-house as described elsewhere [21,22].

### 4.2. Model Heating Experiments with OTA

For the model heating experiments, a stock solution with a concentration of 274 µg/mL in acetonitrile was prepared. Aliquots of the solution (36.5 µL, 10 µg) were transferred into 1.5 mL vials and the solvent was evaporated to dryness under a stream of nitrogen at 40 °C. The dried thin film of OTA was heated for 1–30 min at temperatures of 120, 150, 180, 210, 240, and 260 °C, respectively. Subsequently, the samples were dissolved in 1 mL methanol/water/formic acid (63/37/0.15, (*v*/*v*/*v*)) and analyzed by HPLC-FLD for OTA and 2′R-OTA.

### 4.3. Sample Collection

European coffee samples were obtained from retail markets in France and Germany (and produced by industrial coffee roasting companies). The coffee samples originating from Guatemala were collected from small local markets and produced by local coffee roasters. Cocoa beans and nibs were provided as a gift from August Storck KG, Berlin, Germany. Other food samples reported in Table 1 were commercial products bought from retail markets in Germany.

### 4.4. Sample Preparation

Food samples were analyzed as described in literature with slight modifications [21,38]. To 5.00 g of homogenized ground coffee sample, 100 mL methanol/3% NaHCO_3_-solution (1/1, *v*/*v*) and 50 µL of a solution containing 100 ng/mL d_5_-OTA and 50 ng/mL d_5_-2′R-OTA in methanol were added. The mixture was extracted for three minutes using an Ultra-Turrax T25 mixer (IKA, Staufen, Germany) at a rotation speed of 9500 min^−1^. The obtained suspension was filtered through a 150 mm 3 HW folded filter (Sartorius-Stedim Biotech, Göttingen, Germany) and 5.00 mL of the filtrate were diluted with 45 mL phosphate-buffered saline (PBS) pH 7.4 (8 g NaCl, 1.2 g Na_2_HPO_4_, 0.2 g KCl, and 0.2 g KH_2_PO_4_ dissolved in 1 L H_2_O) before purification using an OchraTest WB (VICAM, available via Klaus Ruttmann, Hamburg, Germany) immunoaffinity column (IAC). After loading the IAC with the sample, the column was washed with 10 mL PBS and 10 mL water. OTA, 2′R-OTA, and DC-OTA were eluted with 2 mL methanol according to the protocol of the IAC manufacturer. The eluate was evaporated to dryness under a stream of nitrogen at 40 °C and reconstituted in 250 µL methanol/water/formic acid (60/40/0.1, *v*/*v*/*v*).

### 4.5. Recovery Rate

For the determination of the recovery rate, a coffee sample containing only traces of OTA and 2′R-OTA was fortified with three different concentrations of OTA (0.5, 2, and 8 µg/kg) and 2′R-OTA (0.4, 0.7, and 1.4 µg/kg) before analysis. To that end, aliquots of a standard solution of OTA and 2′R-OTA in acetonitrile were added to the homogenized sample and the solvent was allowed to evaporate for 2 h before extraction. The recovery rates were determined in duplicate and were 104.4 ± 4.0%, 102.9 ± 4.5%, and 103.9 ± 3.8% for 0.5, 2, and 8 µg/kg OTA and 106.0 ± 0.0%, 109.9 ± 11.2%, and 99.4 ± 3.8% for 0.4, 0.7, and 1.4 µg/kg 2′R-OTA, respectively.

### 4.6. Calibration

For HPLC-MS/MS experiments, an external seven-point calibration of OTA and 2′R-OTA with internal standards d_5_-OTA (1 ng/mL) and d_5_-2′R-OTA (0.5 ng/mL) in a concentration range from 0.1 to 10 ng/mL was used for quantification. The limit of detection (LOD) and the limit of quantification (LOQ) were determined by the signal-to-noise ratios 3 and 9, respectively. For OTA and 2′R-OTA, the LOQ was 0.1 ng/mL and the LOD was 0.03. For the HPLC-FLD experiments, a five-point calibration of OTA, 2′R-OTA (concentration range from 0.10 to 10.0 ng/mL), DC-OTA, and OTα-amide (concentration range from 0.05 to 2.00 ng/mL) was used for quantification. Samples exceeding the calibration range were diluted with methanol/water/formic acid (60/40/0.1, *v*/*v*/*v*) by an appropriate factor and reanalyzed. For all samples, calibration curves with correlation coefficient *r*^2^ > 0.95 were calculated.

### 4.7. HPLC-MS/MS

An Agilent 1100 series HPLC (Agilent Technologies, Waldbronn, Germany) was coupled with an API 4000 QTRAP mass spectrometer (Sciex, Darmstadt, Germany) operated in electrospray ionization (ESI) positive mode with an ionization voltage of 5500 V. Data acquisition and quantification was carried out with the Analyst 1.6.2 software (Sciex). Chromatographic separation was achieved on a Nucleodur C18 Isis (150 × 2.0 mm; 5 µm) column with a 5 x 2 mm guard column of the same material (Macherey-Nagel, Düren, Germany) using a binary gradient at a flow rate of 0.3 mL/min. Methanol containing 0.1% formic acid was used as solvent A and water containing 0.1% formic acid as solvent B. The following linear gradient was used: 0 min 60% A, 1 min 60% A, 10 min 100% A, 10 min 100% A. The injection volume was 50 µL. The mass spectrometer was operated in selected reaction monitoring mode (SRM) with Gas 1 (nebulizer) set to 35 psi and Gas 2 (drying gas) set 350 °C and 45 psi. Nitrogen served as the Curtain gas (20 psi). The following transition reactions were monitored for 100 ms each (declustering potential (DP), collision energy voltage (CE), and collision cell exit potential (CXP) are given in brackets): OTA and 2′R-OTA: quantifier *m*/*z* 404.2→239.1 (DP 60 V, CE 33 V, CXP 14 V) and qualifier *m*/*z* 404.2→221.0 (DP 60 V, CE 50 V, CXP 15 V); d_5_-OTA and d_5_-2′R-OTA: quantifier *m*/*z* 409.2→239.1 (DP 60 V, CE 33 V, CXP 14 V), and quantifier *m*/*z* 409.2→221.0 (DP 60 V, CE 50 V, CXP 15 V).

### 4.8. HPLC-FLD

Samples of the degradation experiments were analyzed with HPLC Germany coupled to a fluorescence detector (X-LC, FP-2020 Plus, Jasco GmbH, Groß-Umstadt). Separation was achieved at 40 °C on a ReproSil-Pur C18-AQ (150 × 4.0 mm; 3 µm) column (Dr. Maisch GmbH, Ammerbuch, Germany) under isocratic conditions using a solvent mixture of methanol/water/formic acid (63/37/0.1, v/v/v) at a flow rate of 0.7 mL/min. The fluorescence detector was operated at an excitation wavelength of 330 nm and emission wavelength of 460 nm. The injection volume was 5 µL.

## Figures and Tables

**Table 1:** Results of a survey screening for the presence of OTA and 2′R-OTA in thermally processed food. (n: numbers of food samples; n.d.: not detectable).

Group	Food Sample		n	OTA (LOD, LOQ)	2′R-OTA (LOD, LOQ)
Cocoa Products	beans (roasted and unroasted)		2	1 sample > LOD	n.d.
	nibs (roasted and unroasted)		2	n.d.	n.d.
	Powder		2	1 sample > LOD	n.d.
	chocolate cream		2	1 sample > LOD	n.d.
Cereals	coffee like	instant malt coffee powder	5	4 samples > LOD 1 sample: 0.62 ± 0.04 µg/kg	3 samples > LOD 1 sample: 0.22 ± 0.02 µg/kg
		malt coffee powder	1	1 sample > LOD	1 sample > LOD
	expanded	puffed wheat	1	n.d.	n.d.
		rye puffed waffles	1	n.d.	n.d.
		rice puffed waffles	2	n.d.	n.d.
	Roasted	popcorn	2	n.d.	n.d.
		Breakfast cereals	2	1 samples > LOD	n.d.
		coloring malt	5	1 samples > LOD	n.d.
	baking	pumpernickel	4	3 samples > LOD 1 sample: 0.11 ± 0.02 µg/kg	1 sample > LOD
